# Electricity in Dental Practice

**Published:** 1911-02-15

**Authors:** L. E. Custer

**Affiliations:** Dayton, Ohio


### ELECTRICITY IN DENTAL PRACTICE.

BY L. E. CUSTER, A.M., D.D.S., DAYTON, OHIO.

In addressing you this evening I am aware that the subject has by this time grown a little old. The time was —and not many years ago—that the dental practitioner was just beginning the use of electricity in his practice. While the subject may not be new, there are applications being made every day that are new, and the field is broadening in every direction. You who live in the city have had these advantages from the very first. I happen to be in a position where I have been able to follow the gradual adoption of this wonderful energy by the profession. It has gradually spread from the city office to the most remote corner of the world, until to-day there is scarcely a dentist practicing this profession but who uses electricity in one form or another. The reason for this is very plain. No profession, science or art has such varied demands for its successful practice as dentistry, and no single agent meets more of these than electrictiy as at present developed.

Reflect for one moment upon the thought that through two wires scarcely larger than a needle can be conveyed into your office a horsepower of energy, an energy with many different properties. It can produce the highest heat known or the most gentle heat for desiccation; it can tear and rip through a rubber plate or it can in the most accurate and delicate manner dress the dentin to the thousandth of an inch from the dental pulp; it can light our offices and it can illuminate the antrum; it heats our office in the winter and cools it in the summer; with it we see through the body, and with the same energy we can build up tissue or we can tear it down. Think for one moment, then, what a wonderful agent we have at our command. Art and science owe their most recent progress to electricity; mechanics would be helpless without it; the successful practice of medicine and surgery can no longer be accomplished without its assistance, and the dental practitioner, more than ever before, finds new uses for this wonderful energy. Not only are many appliances in both branches of dentistry being operated by electricity in one form or other, but the methods of practice are being molded and fashioned by it. While electricity is a most obedient servant, it has built around us such a complete network of wires and devices that we find ourselves servants of electricity. Our obligations to this energy are more than to all other aids and helps combined.

After all these years of study and investigation no one knows what electricity is. Its manifestations are all about us, and its various properties only deepen the mystery. It is an agent in the formation, growth and death of all animal and vegetable organisms. It is the unseen hand at work in nature, the cause of barometric changes, the formidable agent which now and then shatters and tears the atmosphere, terrifying you with the crash of thunder. It transmits the enchantment of a look and the grace of a smile. By some it is regarded a property of all matter; some have thought it to be a vibratory moleculai motion, some a fluid, some two fluids, some a motion in the ether and some the ether itself. We can only study it comparatively. The resemblanec of its phenomena to those of heat and light places it in that class of energy. We have heat, light and electricity derived from friction, from chemical action, from magnetic action and from the sun. Heat produces electricity and electricity produces heat. If the junction of a thermopile be heated a difference of potential will be established, or, if a current be passed through the couplet, the junction will become heated. A crystal of tourmaline and many other crystaline bodies when heated at one end show a difference of potential. Electricity also produces light and will neutralize the polarizing effect of light. Hence from the facts well known as to the nature of heat and light and their reciprocal action with electricity we may infer many of the facts in regard to the nature of electricity. Heat and light are each regarded as a mode of molecular motion, and while electrical phenomena are of great variety and oftentimes present themselves in complicated forms, yet all these, when simplified and understood, show that electrical phenomena are due to an energy not unlike heat and light.

Heat was shown by Davy to be a form of motion in matter and has been so accepted. This motion has been further shown to be vibratory. When a substance conducts heat its molecules are set into vibrations corresponding to the degree of heat, and those substances whose molecules can most easily and quickly assume this molecular agitation are known as good conductors, and those which are slow in so doing are known as poor conductors. It would be fair to suppose, then, that if, as a rule, all those substances which are conductors of heat are also found to be conductors of electricity in a similar degree, the two energizing forces, heat and electricity, would be very similar forms of motion. And so it is found; the degree of conductivity for heat and electricity is nearly the same in like substances. A good conductor of heat is a good conductor of electricity. Light was shown by Fizeau to be a form of motion in ether, and this form of motion has been supposed to be undulatory. It is propagated through the ether in waves similar to the movement of a taut rope when struck. Light will produce electricity and electricity will produce light. A clean metallic plate will become electrified when light falls upon it, or, if the two electrodes of a selenium cell be unequally illuminated, a difference of potential will be felt. This reciprocal action between electricity, heat and light is almost conclusive proof of the theory that electricity is a mode of motion similar to heat and light.

While we do not positively know what electricity is, it has been found, however, to produce results as accurate as those obtained by the application of a rule in mathematics, and when properly understood can be relied upon. A given current flowing through a conductor under a given pressure always produces the same amount of magnetism in an iron core; the same rise in temperature in the conductor, or deposits the same amount of metal from its solution. It is possible for the electrician to calculate beforehand the exact amount of heat that will be produced by the various arrangements of quantity, pressure and resistance. The reliability of the electric current in always producing like results under like conditions is one of the most important virtues of this wonderful agent, especially when used in dental practice, for nearly all dental operations require the utmost accuracy in the application of energy, whether in the form of heat, light, power or chemism.

The perfection and ease in regulating or varying its action for different degrees of strength are important considerations. The speed for revolving the bur, the heat for the desiccation of dentin, for softening gutta-percha, for annealing gold or for fusing porcelain should be to suit each case at hand. These conditions are quite variable, and electricity obeys the throw of the rheostat lever so accurately and quickly that, as if by magic, the will of the operator is felt at the instrument under operation. The electric engine as perfected to-day increases or decreases its speed, stops or reverses at the touch of the operator, and all this is so easily done that after a little use it requires no effort on the part of the operator. The mental routine ceases and the control becomes automatic and the attention of the operator, instead of being divided between the management of the instrument and the operation, may now be concentrated upon the latter.

The use of electricity in the dental office has given dignity to the profession. The supplanting of the pedal engine by the electric engine has done much toward elevating this part of the work from pure mechanical labor. The dentist while operating the pedal engine differed but little from the performance of a scissors grinder in the eyes of the public. The electric engine has added tone to the dental office and dignity to the dentist.

The cleanliness of electricity in every form of its application makes it especially desirable in the dental office, for if there is one place more than another where the most perfect cleanliness should be preserved it is in the dental office. The electric motor may be dust-proof. The incandescent lamp does not consume oxygen nor give off vitiated products. The purity of heat produced in the electric oven or gold annealer is a property not possessed by any other form of heat.

Most of the applications of electricity are noiseless. A horse-power of energy may be coursing through the wires of an electric oven, but there is no sound. The incandescent lamp glows and all galvanic processes go on without audible sound. The best motors can scarcely be heard, and the only sound from electrical processes are those from the sputtering of an arc light due to bad carbons, the little noise of a motor due to bad adjustment of the brushes or the sharp report following a voltaic discharge.

A striking feature of electricity is the flexibility of the system. Anywhere that the two wires can be tapped they will give forth heat, power, light, electrolysis or curative properties, depending upon the instrument or appliance used, and by means of a flexible cord we carry the energy harmless in itself to any part of the office.

The practical applications of electricity in dentistry divide themselves under seven different heads, namely, power, heat, light, electrolysis, cataphoresis, X-ray and electro-therapeutics. We again ponder at the wide range of applications and at an agent which possesess such a variety of properties. It is difficult to comprehend how a single agent should have such manifold properties within itself, an agent that will under one condition melt platinum and under another carry a cocain solution into the dentin; under one condition cause a bur to revolve 4,000 times per minute or under another cause a shaft of light to pass through the body; under one condition it will build up tissue, under another it will tear it down. The electric system of a dental office may be very aptly compared with the nervous system of man. We have the central power house, the brain; the special trunk line, the spinal cord. From that pass out electrical conductors insulated in Neuman's sheath. Nerves for power and motion are distributed to the muscles; nerves for light to the eyes; nerves for electrolysis to the several glands and organs. Every movement in growth and decay is controlled by electrical influences.

Under the head of power we have five practical and useful applications—the dental engine, the lathe, fan, air compressor and electric mallet, and I might add the vacuum cleaner. I need not spend time upon the merits of electrical power, for you must certainly realize that there is no place in all the work of man that the electric motor has a more fitting application. Here again we see the merits which are found in the flexibility of electrical power. I have in my own office, besides motors for engines, lathes, etc., three different electric air compressors, one at eight pounds at the chair for desiccation, one in the laboratory at twelve pounds for soldering and one at forty pounds for casting. The one for desiccation draws its air through a wash bottle of alcohol and ejects it through a heated porcelain tip, and in that manner I obtain a warm—or hot if desirable—jet of air saturated with alcohol, the best drying agent. I should say in passing that the hand piece hangs on a hook, which upon being released starts the motor in operation. This works automatically by taking the instrument up for use. The one for soldering keeps up a steady pressure by an automatic device, while that for casting is put in operation at the time of casting. A quart can is sufficient reservoir, and this is filled in a few seconds, but it cuts off automatically at forty pounds. I might explain in passing that forty pounds may seem to be a high pressure for casting, but with my device I find a high pressure for a moment and gradually decreasing is more certain to cast the gold than an even pressure of twenty pounds.

One of the grand features of electric power is the ease by which a motor of the proper size and capacity can be obtained for each class of work. Instead of running a line shaft for laboratory work, a separate motor is installed for each class of work. I use in my office and residence twenty-four different motors and nearly all are the Emerson, made in St. Louis.

The electric fan as ordinarily supplied is not the best for the operating room. I find that a 12-inch fan motor with a 16-inch blade will give a gentle breeze with a wider spread and without any noise. It should be placed in the left-hand front corner of the room, as far from the patient as possible, and in that way a broader spread of the breeze can be obtained, so that even in the hottest weather this fan cannot be heard.

The electric mallet needs no words of comment where an automatic malleting device of some kind is desired. The operator is relieved of all the work of tripping the device, for here again this subtle agent creeping along the tinsel cords puts life into the magnets at the will of the operator.

We now come to probably the most important and valuable application of electricity in dentistry, namely, heat. These will be taken up in the order of their discovery. The electric annealer is by far the best method of annealing gold for filling. The heat is derived from a platinum wire which is electrically heated. Platinum is a noble metal. It is not oxidized by the heat and it emits neither vapor nor odor; the wire being electrically heated, there is a complete absence of gases, either consumed or unconsumed. The heat of the electric annealer is absolutely pure and the gold annealed thereon is made more cohesive than can possibly be obtained in any other manner. A second advantage is the even annealing of the gold. When the gold is placed upon the electric annealer it is so evenly annealed throughout that no method of flame annealing can compare with it. It is also thoroughly annealed. Being subjected to a heat of 700 degrees Fahr., in a moment it becomes annealed throughout, and it may remain at this heat indefinitely without injury. The cohesive property of gold shows itself at about 250 degrees Fahr., and this cohesiveness increases as the heat is raised. Dr. C. N. Johnson says of this method: "The most perfect method yet suggested is through the medium of the electric gold annealer. * * * With this appliance complete uniformity of result is obtained in the most convenient and ready manner and with no liability of contamination. Even to operators who have been accomplishing satisfactory results by other means, this appliance will soon reveal a working quality to the gold which seems impossible of attainment in any other way; and it is confidently believed that its general adoption by the profession would disarm much of the criticism which is occasionally waged against the manufacturers of gold on the plea of lack of uniformity in preparation." A last point in favor of the electric annealer is the great saving of time. The assistant has only to keep the tray supplied and the operator knows that the gold is properly annealed, and the surface being rough, he becomes so dexterous in picking the gold up that he can do it with the plugger point without change of instruments.

The next form of heat is the electric oven. The almost universal adoption of this instrument immediately after its discovery is sufficient proof of its merit over all other forms of devices or furnaces for fusing dental porcelain. I need not go into the merits of this to any length, for it is conceded that the electric oven is the ideal method of fusing porcelain. The older generation knows with what troubles the fusing of porcelain was fraught. To-day we turn the rheostat lever over to any desired heat—a heat that is the purest at the command of man—and by a clock or the thermostat device the fusing is perfectly done every time. Compare for one moment an oven which you can hold in your hand while it is fusing the highest fusing porcelain to the old coke oven in the cellar or the noisy and unsatisfactory gas or oil furnace. Compare the beauty and uniform product of the electric oven with the uncertain results of other forms, and tell me where in all the applications of electricity it has a more useful field than in the electric oven.

We now come to the melting of platinum. This can be done by using any electric oven as a rheostat and attaching a block of carbon to one terminal and an electric light carbon to the other. Platinum in small quantities can be melted if placed in the arc formed between the carbon plate and pencil when they are separated. The electric arc is estimated to be 6000 degrees Fahr. Platinum melted in the presence of carbon becomes quite stiff and is a useful substitute for platino-iridium. If we wish our platinum to be soft, use a block of lime to place the platinum upon and play upon this with a platinum-pointed electrode. In this way the platinum will be as soft as new.

No operation in dentistry requires the nice regulation of heat that the desiccation of sensitive dentin does, and here again electricity stands out as pre-eminently the best. The electric heat of the warm air syringe can be regulated most perfectly to any desired degree. I should state that while nearly all forms of warm air appliances are good, absolute uniformity of heat can only be had in an appliance in which the heat is generated in the syringe nozzle itself. This was pointed out by the lamented Dr. Wassell. It is impossible to conduct heated air through a rubber tube with uniform results. In order that the air be of a uniform temperature the pressure must be fixed, as well as the heat of the wire over which the air passes. Varying either of these conditions will modify the temperature of the blast.

A fourth form of heat is the cautery. You are familiar with this and its uses, and I only need call your attention that here again electric heat has its advantages over any other form.

The electric heater for gutta-percha and instruments for its working gives more uniform results than any other device.

The electric sterilizer is the neatest method of sterilizing by heat, and we as dentists should use more heat in sterilization. We should bear in mind that many of the most poisonous germs are surrounded by a capsule which only heat can penetrate.

The electric warm water device is the neatest for that purpose. Many such are on the market, but I believe we have been doing this "wrong end to," so to speak. Heretofore we have been heating the water in a vessel and drawing it into a cold bulb. I have made a porcelain receptacle for the bulb, which slips on the edge of the glass. This receptacle is electrically heated. In this way I heat the water in the bulb and not in the glass. The resistance for this heater is an eight candle power lamp, which is incidentally used as a heater for inlay wax, after the method used by Dr. Taggart. A glass tray is placed at such a distance above the lamp as to give the proper heat for softening the wax. In this manner we have two splendid applications of electricity in the most economical way.

One of the most essential features in the casting of a gold filling is perfect fluidity of the gold. If the arc light is used for this purpose, we have an agent twice as hot as the oxyhydrogen blowpipe and the gold can soon be brought to the boiling point. So here again we find the use of electricity creeping into every substantial improvement in dentistry.

The last application of heat to which I will call your attention is a simple yet useful device. Wherever I have a gas jet or blowpipe I have what I call an electric match. This consists of a carbon or platinum-tipped device like a tuning fork. When the ends are pressed together and then released a small arc is formed sufficient to light escaping gas. A 32 c. p. lamp, an electric motor or annealer will serve as a rheostat.

No dental office is complete without electric light. The ordinary gas jet gives but a limited amount of light. It robs the air of its oxygen and it fills it with life-killing residue. The electric lamp is hygienic, and instead of a light of but one strength we can have any desired strength. The fixtures are ornamental and our offices are beautified by the electric lamp. The electric mouth lamp is a gem in itself. The one recently devised by Dr. David Stearn is not only with little heat, but it is so strong that in many cases it takes the place of an X-ray exposure. This light is contained in a surrounding vacuum shield, which, like a thermos bottle, does not conduct the heat.

Under the head of electrolysis we have the electroplating of our instruments and regulating appliances. This can easily be done by the dentist himself from the 110-volt direct current. Copper amalgam is easily made by electrically depositing copper into the mercury. The worst

"roasting" a dentist ever got was from your honored Dr. McKellops upon myself when I described this method at the old Mississippi Valley Dental Society.

When considered strictly from a scientific point of view cataphoresis was the surest and best method of anesthetizing dentin and the dental pulp ever proposed. Unfortunately, this operation was filled with so many details, the failure of any one of which meant a failure in the end, that it soon fell into disuse. It was an educator, however, for to-day we have high pressure anesthesia, which is a mechanical way of producing cataphoresis. Dr. Jack truthfully says, "When the pain attending excavation requires active treatment, such as the employment of zinc chlorid or general anesthesia, the cataphoric method is far preferable to either and is absolutely certain of giving relief. The results of successful cataphoresis are marvelous, and it may be truly stated that no advance in years in the therapeutic treatment of teeth is comparable to this."

It will always stand to the credit of dentistry that the dentist was foremost in the practical application of the X-ray. Drs. Kells, Price, Van Woert and others almost immediately—in fact, just as soon as the instruments were available—established it in their practice. Not only that, but they were instrumental in many of the improvements in the appliances and technic of operation. While it is not practical for every dentist to own an appliance, no dentist is up to date who has not access to a specialist. Stearn's lamp is a great aid in diagnosis, but the X-ray is the only positive diagnostic agent in foreign bodies, unerupted teeth, abscesses and conditions of the antrum.

Of electro-therapeutics proper not much more can be said than has been said under the head of electrolysis, cataphoresis and the X-ray. The X-ray, by reason of its therapeutic effects in many cutaneous diseases, has been tried for pyorrhea, but the dangers attending its prolonged administration necessary in those cases do not warrant its general use. Dr. Ebersole, of Cleveland, has been using and advocating the violet ray for neuralgic conditions. The violet ray is next in order in rate of vibration to the X-ray, and since it is well established that the X-ray has unquestionable therapeutic value, we believe it is not beyond the power of the violet ray to produce results of a like but milder character. For this reason the violet ray may find a practical place in dentistry.

On summing up you will be surprised to find that I have shown twenty-four different uses for the electric current in dental practice at the present time, and it stands to the great honor and credit of the American dentist that with the single exception of the X-ray all are American inventions; and I may say in conclusion that the future progress of dentistry will be largely based upon the unfoldings of electricity, which to-day have only begun.—*Dental Brief.*

